# Automatic Couinaud segmentation using AI and pictorial representation landmarking

**DOI:** 10.1007/s00261-025-05123-3

**Published:** 2025-07-30

**Authors:** Luis Miguel Núñez, Paul Aljabar, Sir Michael Brady

**Affiliations:** Perspectum Ltd, Oxford, United Kingdom

## Abstract

**Objectives:**

Delineating the Couinaud segments is a critical component of liver surgery and monitoring that has traditionally relied on labor-intensive methods that are prone to variability. While fully or semi-automatic methods exist, they generally lack accuracy or require extensive post-processing or corrections to the outputs.

**Methods:**

We present a framework that integrates deep learning-based segmentation with auxiliary landmark identification to create a personalized pictorial model on which to base precise Couinaud landmark localization. Data from 225 non-contrast T1-weighted MRIs from 4 different studies were used to evaluate the performance against benchmark techniques and human-defined ground truth.

**Results:**

The personalized model outperformed the benchmark method in every landmark placement and Couinaud segment volume estimation, being significantly better in 5/8 landmarks and 7/8 segments.

**Conclusion:**

The proposed system is explainable, agnostic to imaging modality and is able to incorporate new data without retraining, enhancing its robustness and scalability across diverse clinical contexts. These findings underscore the potential of our framework to substantially improve Couinaud accuracy and streamline clinical workflows, optimizing liver surgery planning and monitoring.

**Supplementary Information:**

The online version contains supplementary material available at 10.1007/s00261-025-05123-3.

## Introduction

Couinaud segments are the most commonly used anatomical division of the liver for detection and monitoring of hepatic lesions and liver surgery [1, 2]. Using the vasculature as a reference, the method parcellates the liver into nine non-overlapping segments. In liver resection, the future liver remnant that a surgeon will leave intact after surgery depends on the health of the liver parenchyma, and its accurate measurement is key for a successful intervention [3, 4]. Couinaud segments have been adopted as a standard in surgery to support decisions on those parts of the liver that may be resected [5] so again accurate parcellation of the Couinaud segments is needed in order to predict segment volumes.

Manual delineation of liver Couinaud segments in 3D images, typically CT or MRI, is both laborious and time-consuming, motivating the development of automatic or semi-automatic methods. Several such methods have been developed recently. For example, semi-automatic approaches were developed using region-growing [6] and mixture modelling [7]. Fully automatic deep learning based delineations have also been developed [8]. Current methods, however, remain limited in either requiring manual inputs to initialize the delineation or needing extensive post-processing or correction in the case of sub-optimal outputs. The work of Arya et al. [9] offers a solution that seeks to address both issues, performing Couinaud segmentation based on a set of anatomical landmarks in the vasculature and other structures of interest that divide the liver along subsequently defined planes, where landmarks are found automatically, and any correction is reduced to the relatively easier step of shifting landmarks to an improved location. However, this approach lacks explainability, as it is not clear how the model determines the landmark positions or generates heatmaps, despite these being supposedly related to specific anatomical structures. This lack of transparency in the decision-making process of the model limits its interpretability and potentially its clinical acceptance.

Pictorial structure representations for objects, first developed in 1973 [10], combine several components of the expected structure which are inter-connected in a deformable configuration. This configuration enables inference of unknown components using data and measurements of observed components and the pictorial representation of the whole object. This technique has been used in studies of face detection [11], human position detection [12, 13] and has been extended to the field of medical imaging [14, 15] by assuming anatomical parts of the body to have variable but generalisable modelling the spatial relationships of anatomical parts of the body. For example, the number of ribs is generally predictable, as is the structure of the spine; but the lengths, thicknesses, and separations of the ribs varies between individuals, as does the distance from sternum to the spine, in ways that can be represented with statistical distributions.

In this study, we propose a novel method that combines the performance and robustness of deep-learning-based segmentation models and the explainability of pictorial representations to provide robust and explainable automatic Couinaud segmentation. Our approach outperforms state-of-the-art automatic landmarking systems while offering enhanced interpretability and reliability. The method involves constructing a pictorial configuration model based on landmarks, which includes both those directly used for Couinaud segmentation as well as an additional set of “auxiliary” landmarks. A subset of these landmarks that are well-predicted from features of deep learning-based segmentations is used in conjunction with the pictorial configuration model to predict the locations of the remaining Couinaud landmarks. Our method is evaluated against a state-of-the-art automatic landmarking method and the observed variability in expert annotations.

## Methods

### Couinaud segmentation

**Fig. 1:**
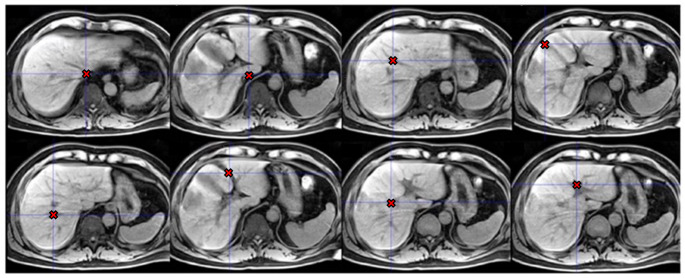
Images of examples of Couinaud landmarks. Left to right, top row: inferior vena cava inferior, inferior vena cava superior, middle hepatic vein, gallbladder fossa. Bottom row: right hepatic vein, umbilical fissure, right portal vein, left portal vein

We divide the liver into nine Couinaud segments by specifying planes that are defined by the locations of eight anatomical landmarks, as described in the guide by Germain et al. [2]. The specific landmarks we use are: inferior vena cava inferior (IVCi), inferior vena cava superior (IVCs), gallbladder fossa (GBF), umbilical fissure (UF), left portal vein (LPV), right portal vein (RPV), middle hepatic vein (MHV) and right hepatica vein (RHV). These landmarks are illustrated in Fig. 1. Planes dividing the liver are then constructed from subsets of three landmarks. Only one segment, the caudate (Segment 1), is not defined based on the landmarks and performance metrics for this segment are excluded from the results, as it is not affected by landmarking accuracy. We note that in clinical practice, Couinaud segments are defined by vascular flows, and the boundaries between them are not in general planar. However, the planes we propose serve to approximate these boundaries based on guidelines that enable more precise localisation for surgery.

### Auxiliary landmarks

The Couinaud segments are modelled using a pictorial representation of elements that are defined by the positions of landmarks, and the configuration is determined by the displacements between the landmarks in three dimensions. To enrich the model, as well as those directly used to define the Couinaud segments, additional *auxiliary landmarks* are incorporated which are located in and around the liver.

A total of nine auxiliary landmarks were defined using heuristics on the structures delineated by Total Segmentator [16, 17], a robust deep-learning model that delineates up to 59 different structures over the entire body. Anatomical parts in and around the liver delineated by this model are used to extract the landmarks and define the pictorial model of this area. These structures were the liver, right kidney, pancreas, portal vein, gallbladder, inferior vena cava and heart. Details and example images of each of the auxiliary landmarks are shown in the supplementary material.

Auxiliary landmarks can be viewed as defining a framework around the liver that constrains the locations of the landmarks that we aim to predict, and which will define the Couinaud planes. It is important for the auxiliary landmarks to be anatomically meaningful and easy extract reliably from the outputs provided by the segmentation model (Total Segmentator in our study). In essence we deploy them on the grounds that if we know the locations of organs such as the kidneys, heart, and gall bladder, we can constrain the location of the liver and structures such as vena cava.

### Pictorial structures configuration

**Fig. 2:**
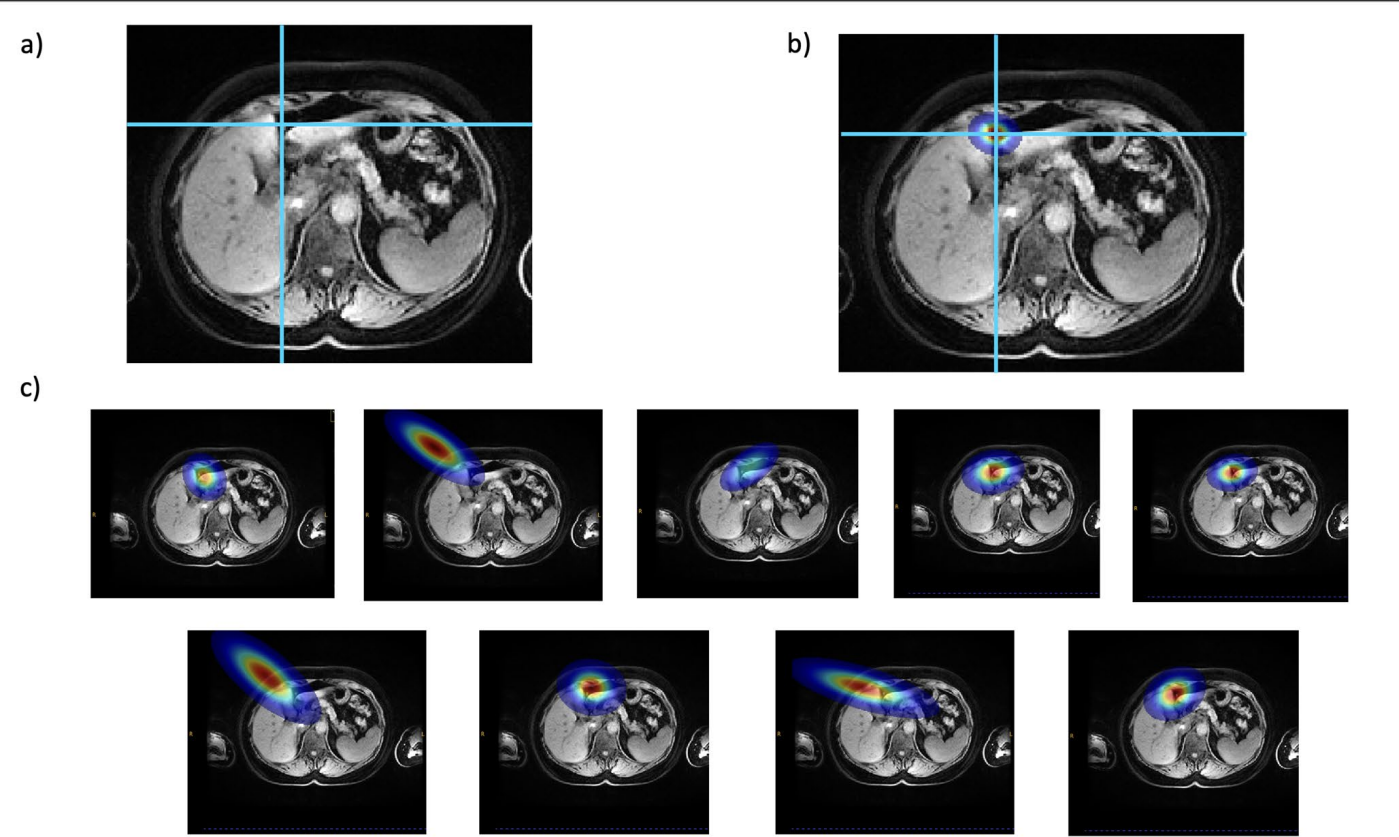
Example of landmarks probability heatmaps for umbilical fissure (UF) and the contributions of each known landmark. **a** Raw T1w image, dashed blue lines represent the location of the predicted landmark. **b** Combined heatmap used to place the landmark from the combination of all individual heatmaps. **c** individual probability heatmaps. Left to right, top to bottom. Top of gallbladder; bottom right of heart; Liver bottom right; Liver center; Liver left corner; Pancreas top right; Top of right kidney, top of portal vein and Liver top right

The configuration model is defined by a four-dimensional data matrix of the displacements in 3D between each pair of landmarks for each scan. The data matrix has size $\:P\times\:N\times\:N\times\:3$, where *P* is the number of patients, and *N* is the number of landmarks and is similar to the spatial model described by Potešil et al. [18]. The model uses displacements between pairs of landmarks instead of locations as the important information is encoded in their relationships rather than in their specific positions. To estimate the position of an unknown landmark $\:u$, a spatial probability map $\:{P}_{u}\left(v\right)$ is estimated, and the voxel location $\:v$ with highest probability is assigned to the landmark. The probability map, $\:{P}_{u}$, is estimated by multiplying the individual conditional probability maps $\:{P}_{u,\:k\:}$(examples shown in Fig. 2), where $\:k$ ranges over the $K$ *known* landmarks, each represented by a 3D Gaussian with mean displacement $\:{\mu\:}_{u,\:k}$ and covariance matrix $\:{\varSigma\:}_{u,\:k}$ derived from the 3D displacements between landmarks $\:u$ and $\:k$ in the training set. Missing landmarks in training cases are not included in the calculation of the mean and covariance of 3D Gaussian displacements:    

$$\:{P}_{u}\left(v\:\right)=\:\prod\:_{k=1}^{K}{P}_{u,\:k}\left(v\:|\:{l}_{k}\right),$$

$$\begin{aligned} &{P}_{u,k}\left(v\:|\:{{l}_{k}\:;\:\mu\:}_{u,\:k}\:,\:{\varSigma\:}_{u,\:k}\right) \\ &=\:\frac{1}{{\left(2\pi\:\right)}^{\frac{3}{2}}\:{\left|{\varSigma}_{u,\:k}\right|}^{\frac{1}{2}}\:}\:{e}^{\left({-{\frac{1}{2}}{(v-{l}_{k}-\:{\mu\:}_{u,\:k})}^{T}\:{{\varSigma}_{u,\:k}}^{-1}(v-{l}_{k}-\:{\mu\:}_{u,\:k})} \right)}.\end{aligned}$$

A pictorial model created from a large cohort creates probability distributions for the displacements between pairs of landmarks that tend towards the representation of an ‘average person’ over the population. However, landmark distribution of some patients may not be well approximated by this average distribution. There may be idiosyncrasies for the individual case, for example if they have previously undergone resection, if there are tumours that distort organ shapes, or even an abnormal patient size and organ distribution, making the population average a suboptimal representation to model landmark distribution. To mitigate this, we create a personalized pictorial model for each patient by selecting a subset of the most anatomically similar cases from the full training set. Similarity is assessed based on the configuration of *known* landmarks: For anatomically similar patients, we expect a similar pattern of corresponding displacements between the known landmark pairs. The selected subset is then used in estimation of the spatial probability maps for the missing landmarks as described earlier. For a heterogenous population, where different anatomical variants may be present, the subset selection method provides an estimate of distributions of sub-groups, as encoded in the data matrix and derived mean and covariance matrices.

The distance, $\:{D}_{{p}_{A},\:\:{p}_{B}}$, between two patients, A and B, is estimated by the sum of the absolute differences in displacements for all $\:K$ known landmarks. If a landmark is missing in either patient A or B, the landmark is removed from the set of landmarks used in the distance calculation. The personalized model trained in the same way as the original one, with just a change the cases selected from the training set.

$$\:{D}_{{p}_{A},\:\:{p}_{B}}\:=\:\frac{\sum\:_{{k}_{1}=1}^{K}\sum\:_{{k}_{2}=1}^{K}\:\:\left|\left({\boldsymbol{l}}_{{p}_{B},{k}_{1}}-\:{\boldsymbol{l}}_{{p}_{B},{k}_{2}}\right)-\left({\boldsymbol{l}}_{{p}_{A},{k}_{1}}-\:{\boldsymbol{l}}_{{p}_{A},{k}_{2}}\right)\right|}{{K}^{2}}.$$

### Data and Experiments

The analysis used non-contrast T1w MRI datasets for 225 patients from several clinical trials comprising scans from several clinical trials, including Precision1 (NCT04597710), HepaT1ca (IRAS 223180) and two other ethically approved studies (IRAS IDs 213343 and 226607). The full cohort contains scans of patients without liver resections prior to the image acquisition and with different diseases acquired using Siemens, Philips, and GE scanners, at field strengths of 1.5T and 3T. Couinaud landmarks were manually placed for all images by experienced operators, and these were considered to be “ground-truth”.

We compared our method against the algorithm developed by Arya et al. [9], which is treated as a benchmark for estimating the Couinaud segmentation for the same set of landmarks. For each method, we calculated the distances between predicted and manually defined Couinaud landmarks. Couinaud delineations defined by the landmarks from each automated method were also assessed against the ground-truth in terms of the percentage of the whole liver volume to assess the potential effect on liver surgery planning, where the percentages determine thresholds for surgical decisions.

Of the 225 images used in creating the pictorial representation model, none were used in the development of Total-Segmentator and 99 were used in the training or validation of the benchmark model. The 99 cases were removed from the test set to avoid overfitting biases in the experimental results, leaving a final test set of 126 cases. The pictorial configuration model allows for leave-one-out-cross-validation to be performed, predicting each case with a model made by the 224 remaining cases by simply removing a target case’s submatrix from the 4D model matrix.

When building personalized pictorial models, the number of similar cases selected used to form the training set was varied to assess its effect on the model performance.

The outputs of new and benchmark methods were also compared against the inter-operator experiment performed by Mojtahed et al. [19] for ten cases from another ethically approved cohort (IRAS ID 241312) to compare the variability of the proposed method with the variability observed when manually creating the ground-truth data.

## Results

**Fig. 3:**
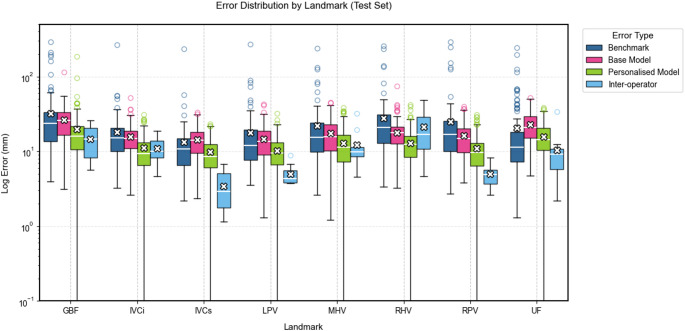
Log error in landmark location for each method. The box plots compare four different approaches across each landmark. The plots show the median (horizontal line within boxes), interquartile range (boxes), and outliers (individual points) of error measurements. The white crosses indicate mean values for each method. Lower error values represent better performance

**Fig. 4:**
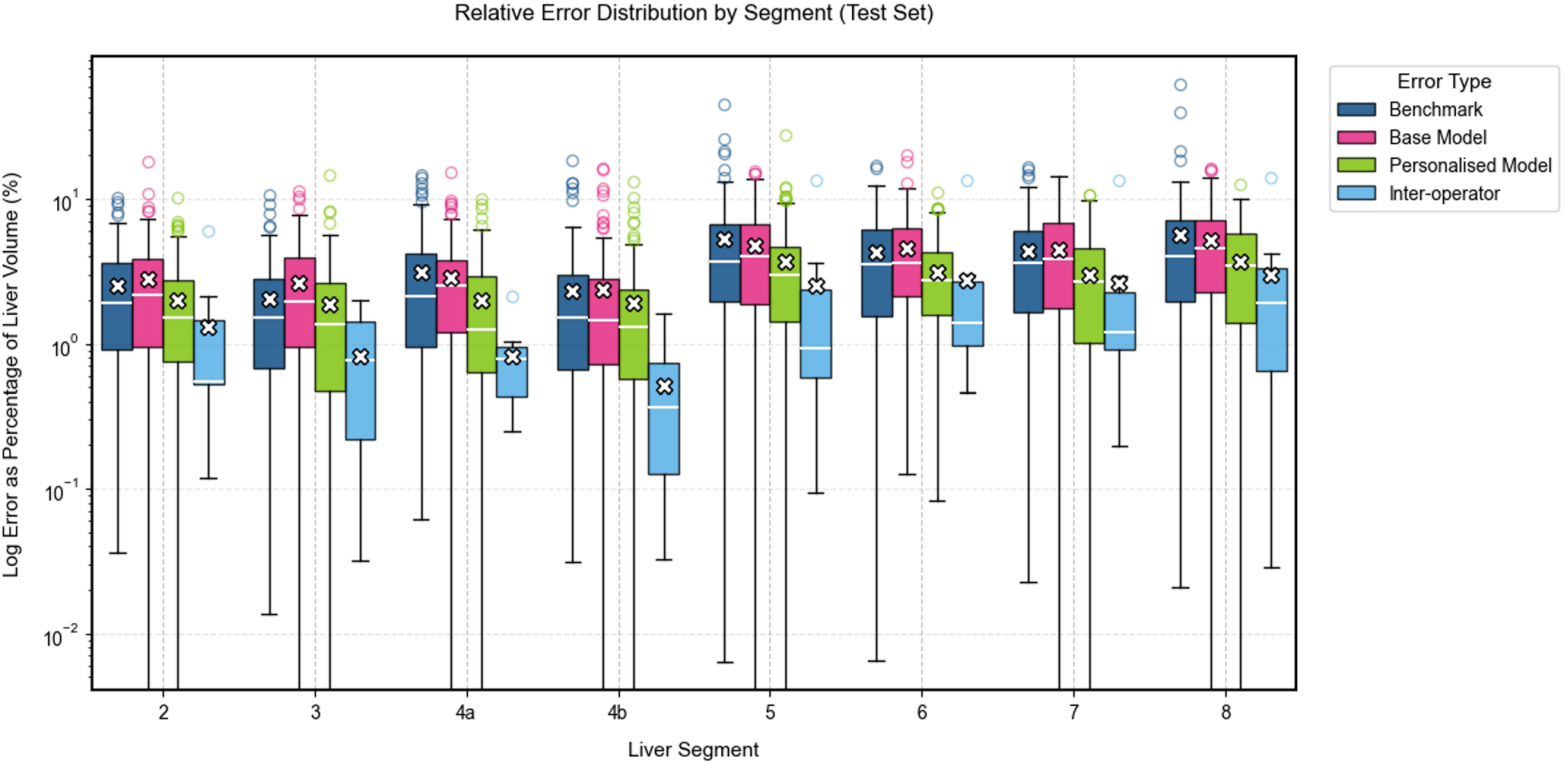
Log error in Couinaud segment volume for each method. The box plots compare four different approaches across each landmark. The plots show the median (horizontal line within boxes), interquartile range (boxes), and outliers (individual points) of error measurements. The white crosses indicate mean values for each method. Lower error values represent better performance

The 126 test set images, selected from the original 225 images used to construct the pictorial model, were analysed using both the proposed and benchmark method. The distances between the predicted landmarks and the ground-truth landmarks were calculated and compared (Fig. 3) alongside inter-operator distances for additional context. Furthermore, the variation in Couinaud segment volumes relative to the ground truth was assessed and compared across methods (Fig. 4).

Within the dataset used to build the pictorial model, 40 patients had one missing landmark, and 3 patients had two missing landmarks. These missing landmarks were excluded from the Couinaud landmark estimations of patient similarity metrics. Their absence was attributed to several factors, including model underperformance, anatomical absence of the target structure, or poor image quality—such as noise or artifacts—that impeded accurate visualization.

The base pictorial model (without personalization) outperformed the benchmark method in landmark localization for most landmarks (Table 1), with statistically significant improvements for RHV and RPV (*p* = 0.002 and *p* = 0.012). However, it yielded significantly higher volume error for segment 3 compared to the benchmark (Table 2).

**Table 1:**
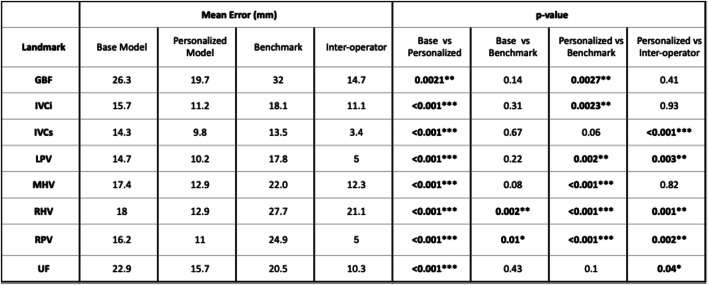
Landmark error comparisons

**Table 2:**
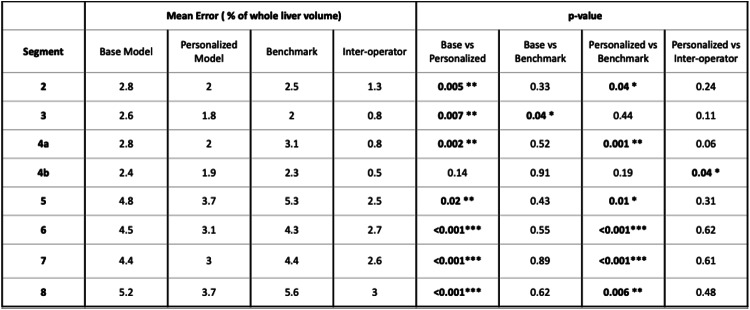
Couinaud segments volume error comparisons

**Fig. 5:**
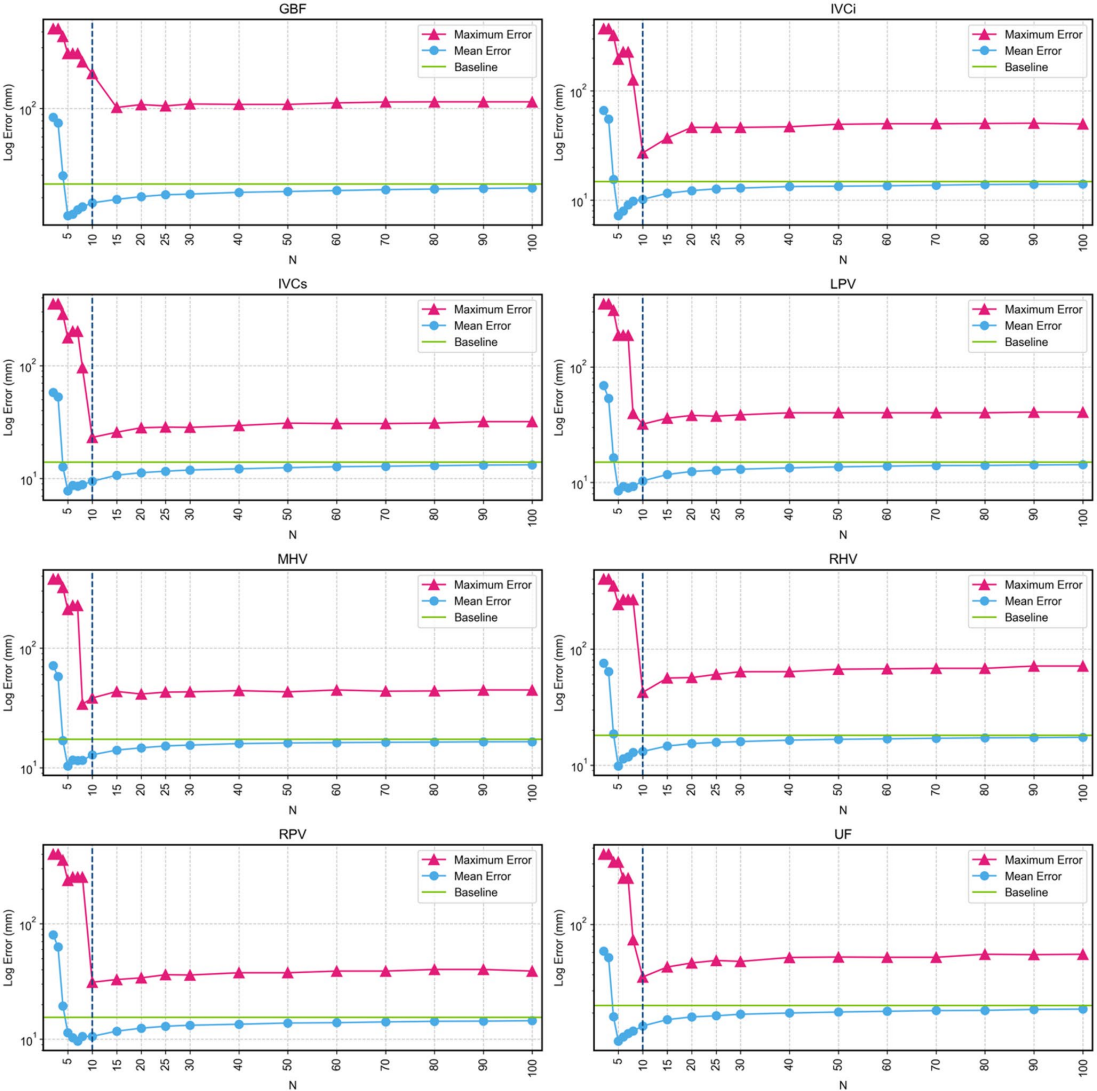
Maximum and mean log error for each landmark as a function of the number of training samples (N). Pink line represents maximum error, light blue line represents mean error, green line represents the mean error at baseline, and the vertical dashed line shows the selected N (10) for the experiments when assessing personalized the model

For the personalized pictorial model, different sizes of training set were evaluated to identify the optimal configuration that improves model performance (Fig. 5). In order to identify an accurate and robust model the personalized model, both maximum and mean error were assessed to decide on a good subset size. Ultimately, a subset of size ten was selected as it gave the lowest maximum error for the majority of landmarks while having a low mean error, showing both accuracy and robustness. All the experiments for the personalized model were then conducted with this subset size.

Compared to the benchmark method, the personalized model showed statistically significant improvements in six out of eight landmarks (GBF, IVCi, LPV, MHV, RHV, RPV) and five out of eight Couinaud segments (3, 5a, 6, 7, 8).

When compared against inter-operator variability, the personalized model showed significantly higher error in five landmarks (IVCs, LPV, RHV, RPV, and UF), and in segment 4b for volume estimation.

The percentage of cases with at least one or more segments with a volume error above 10% was calculated. On this metric, the personalized model (9.8%) outperformed the benchmark model (31.7%) and the base model (30.1%). It also outperformed the inter-operator variability where 10% of cases had a variability of 10% or more in at least one segment.

## Discussion

Our method automates Couinaud segmentation by combining deep learning models and pictorial representations of anatomical landmarks within a single framework. Beyond improving landmark prediction accuracy, the method enhances reliability and robustness by substantially reducing the likelihood of implausible or outlier landmark suggestions. The combination of individual probability maps from multiple auxiliary landmarks provides complementary spatial context, contributing to more consistent and trustworthy results compared to the benchmark approach.

Personalized pictorial modelling, by using known landmarks to select a subset of the training data, gave excellent results in landmark localization. Interestingly, as the number of cases used in the personalized training set decreases, performance improves until a threshold is reached at approximately *N* = 10 (Fig. 5). Below this point, the model becomes unstable and there is an increase in outliers despite improved overall mean performance. After a further decrease, around *N* = 4, performance diminishes due to a poor pictorial representation with such a small sample. Although the specific threshold values varied, the pattern of performance changes with decreasing subset samples remained consistent across all landmarks.

The base pictorial model exhibited strong performance in locating landmarks and measuring Couinaud segments volumes, comparable to the benchmark model in seven out of eight metrics, both for landmarks and segment volumes. Notably, performance was significantly enhanced when using a personalized training set of ten cases. This approach outperformed the benchmark method in six out of eight landmarks and five out of eight segments, with four of those segments contributing most significantly to the overall liver volume. These are also the segments where mis-segmentation could critically impact surgical planning. Furthermore, when compared to inter-operator variability, only one segment demonstrated a significantly higher error with the personalized method.

The variations in error levels across landmarks, when compared to inter-operator variability, may be explained by two main factors: some landmarks may naturally have greater anatomical variability, making their positions harder to infer from surrounding structures, and the inherent difficulty for human operators to visually identify these landmarks consistently during manual annotation. The personalized model also displayed robust performance, with only 9.8% of cases exhibiting at least one segment with more than 10% error—comparable to inter-operator variability and a marked improvement over the base and benchmark methods. In instances where a reviewer deems a landmark suboptimal, like in those significantly worse than inter-operator variability, corrections are as straightforward as adjusting the landmark to its optimal position with a single click.

Beyond performance metrics, the pictorial model offers several advantages over conventional deep-learning approaches. In terms of explainability, unlike black-box models, where internal decision-making is often opaque, our method relies on interpretable geometric relationships between anatomical landmarks. The data used for landmark prediction correspond to actual physical displacements observed in a cohort of patients, grounding the model in measurable anatomical variability. Furthermore, as illustrated in Fig. 2, the final landmark heatmap can be decomposed into individual components, where the influence of each known landmark is explicitly represented through its own weighted probability map. This transparent structure enables clinicians and researchers to trace the evidence for each prediction.

Since the pictorial configuration model only contains the real-world distances between anatomical landmarks in the training set, it remains independent of imaging modality or sequence type. This makes it readily extensible to new imaging data, provided the corresponding segmentation models perform adequately. In our example, the Total-Segmentator software supports both CT and a range of MR sequences. This flexibility should facilitate the accumulation of a larger, modality-independent training set, allowing for better personalized modelling as the dataset grows. Despite that, Couinaud segment predictions remains fundamentally dependent on the quality of the initial segmentation. Errors in upstream segmentation (due to low image quality, anatomical anomalies, or modality-specific limitations) can propagate to landmark detection. Additionally, variations in imaging sequence, contrast enhancement, or spatial resolution may introduce systematic biases in the placement of both auxiliary and Couinaud landmarks, particularly if such variability is not adequately represented in the training data.

The pictorial model also acts as a compact representation, while the personalized set selection method functions like a database query. This approach is particularly useful in cases where certain landmarks are missing or incorrectly placed, such as with resected gallbladders. Adding new data to the pictorial model is straightforward: inter-landmark distances (after manual correction) can simply be appended to the model matrix. This eliminates the need for the time-consuming and computationally expensive retraining process required by deep-learning approaches when updating training data [9]. However, the current model was trained exclusively on patients without prior liver resections or significant anatomical alterations. As a result, its generalizability to complex clinical presentations—such as altered liver morphology due to surgery, pathology, or congenital variants—is currently limited. We would expect a drop in performance when applied to such cases, given that their anatomical configurations fall outside the learned variability. To improve robustness, the model should incorporate a broader range of anatomies, allowing the personalized selection mechanism to retrieve more relevant training analogues during inference.

Future work should prioritize inclusion of multimodal and pathological datasets, more extensive clinical validation across diverse surgical scenarios, and integrated assessments of segmentation quality to quantify its effect on landmark accuracy.

In summary, this study demonstrates that combining deep learning with a pictorial model of anatomical landmarks provides a robust and efficient framework for segmentation of the Couinaud segments. The approach enhances accuracy, improves explainability, and offers flexibility across imaging modalities while addressing critical issues such as data privacy and inter-operator variability. The ability to readily integrate new data without retraining further underscores its scalability. These features make this method a promising tool for advancing liver segmentation and improving its utility in clinical applications such as surgical planning and outcome prediction.

## Electronic supplementary material

Below is the link to the electronic supplementary material.

Supplementary Material 1

## Data Availability

No datasets were generated or analysed during the current study.
