# Supplementary material for: Automatic Couinaud segmentation using AI and pictorial representation landmarking
Source: Abdom Radiol (NY). 2025 Jul 30;51(3):1586–94. doi: 10.1007/s00261-025-05123-3 (PMC12971927; doi:10.1007/s00261-025-05123-3)

SUPPLEMENTARY MATERIAL

Automatic Couinaud segmentation using AI and pictorial representation based landmarking.

In our study a pictorial configuration model is created from a set of landmarks including those used for the Couinaud segmentation and a set of auxiliary landmarks. In this appendix we show the auxiliary landmarks used in the model on how they were defined using the outputs from the Total-Segmentator tool.

- **Liver Bottom Right**: Found on the most inferior axial slice containing liver tissue. The algorithm locates the rightmost point of the liver contour on this slice.
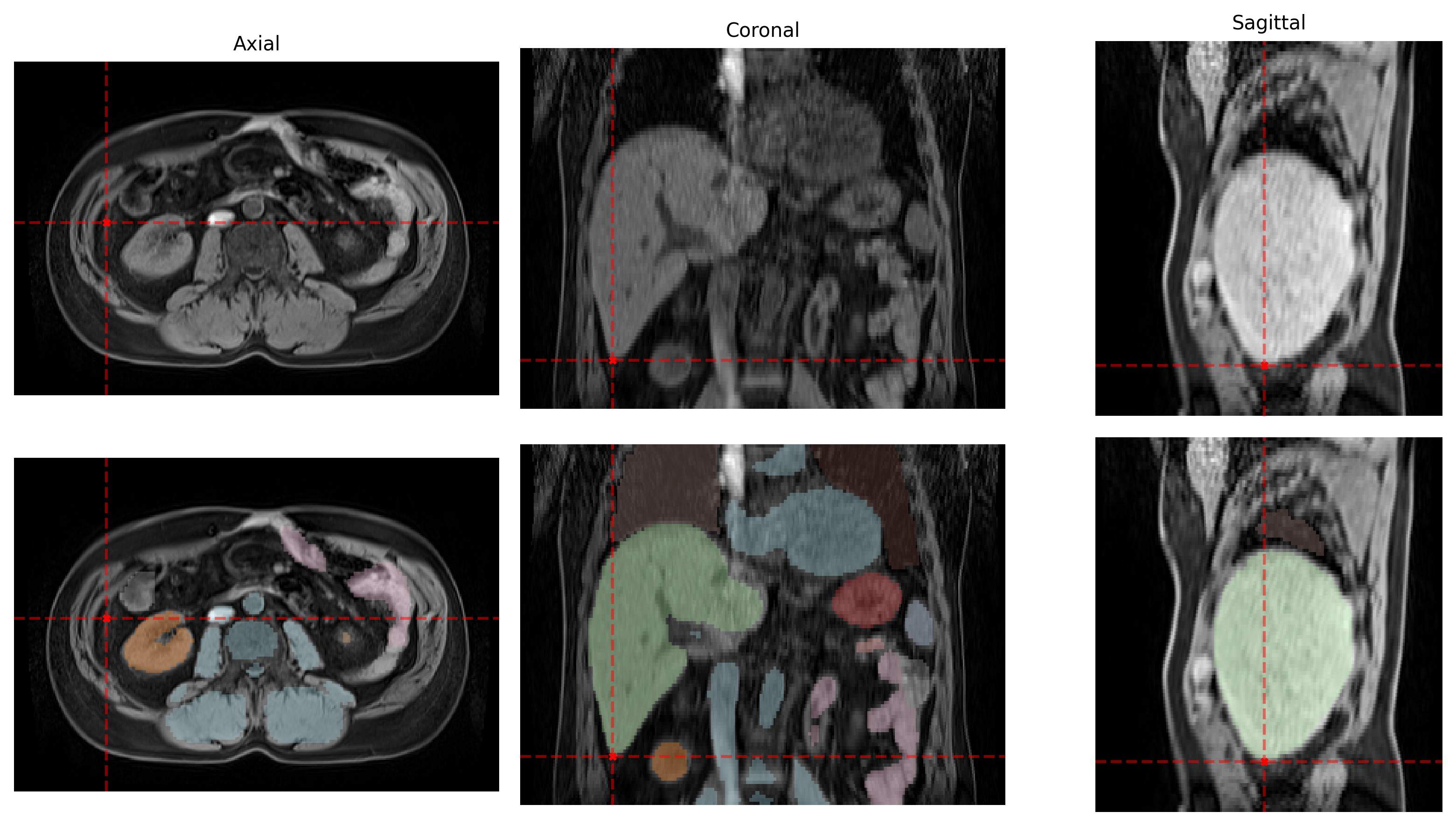

- **Liver Center**: Computed as the centre of mass of the entire liver segmentation mask using all three dimensions.


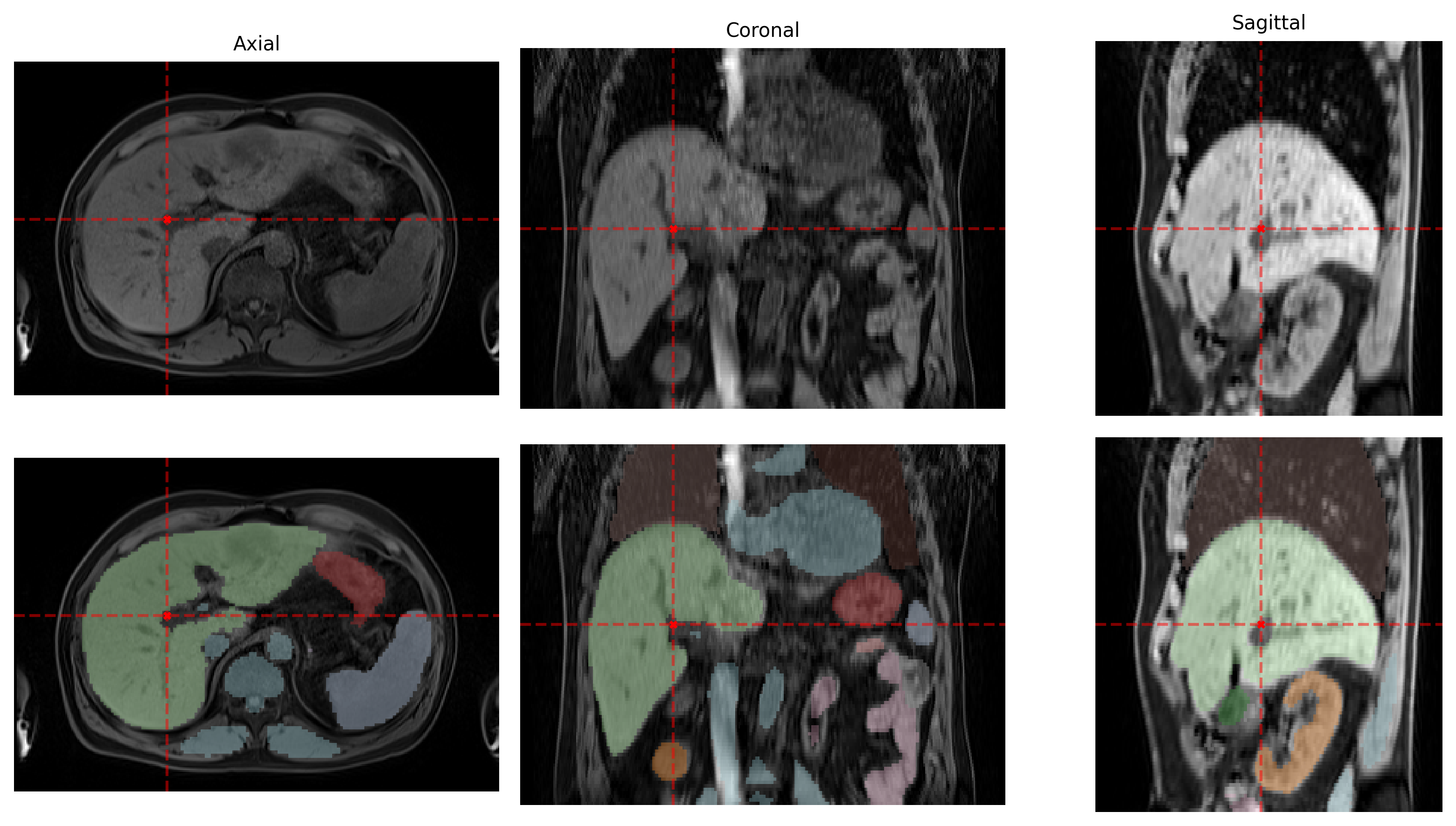


- **Liver Left Corner**: Determined by first finding the leftmost sagittal slice containing liver tissue, then identifying the superior point of the liver contour within this slice.
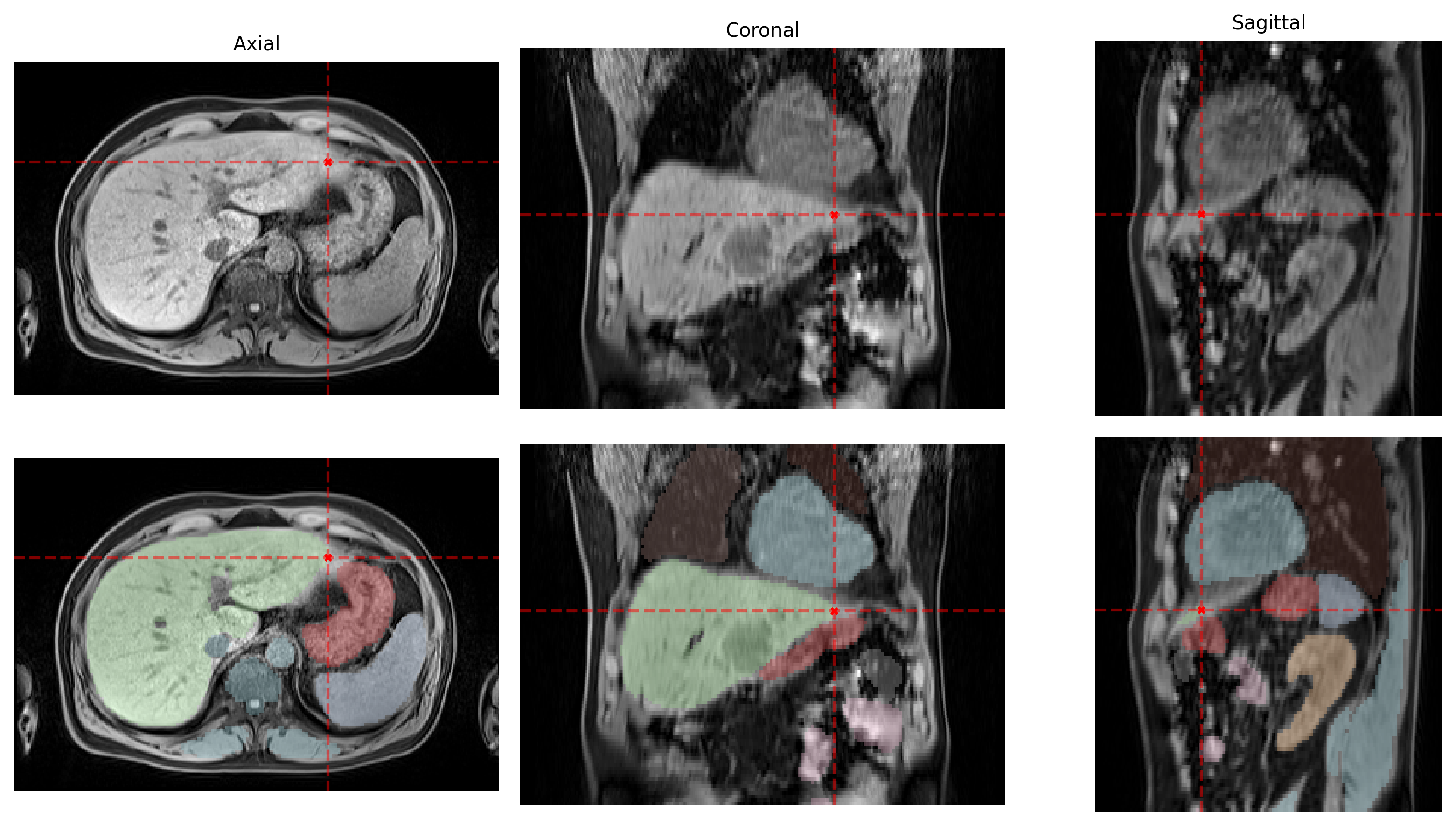

- **Liver Top Right**: Located on the most superior axial slice containing liver tissue. Within this slice, the algorithm identifies the rightmost point of the liver contour.
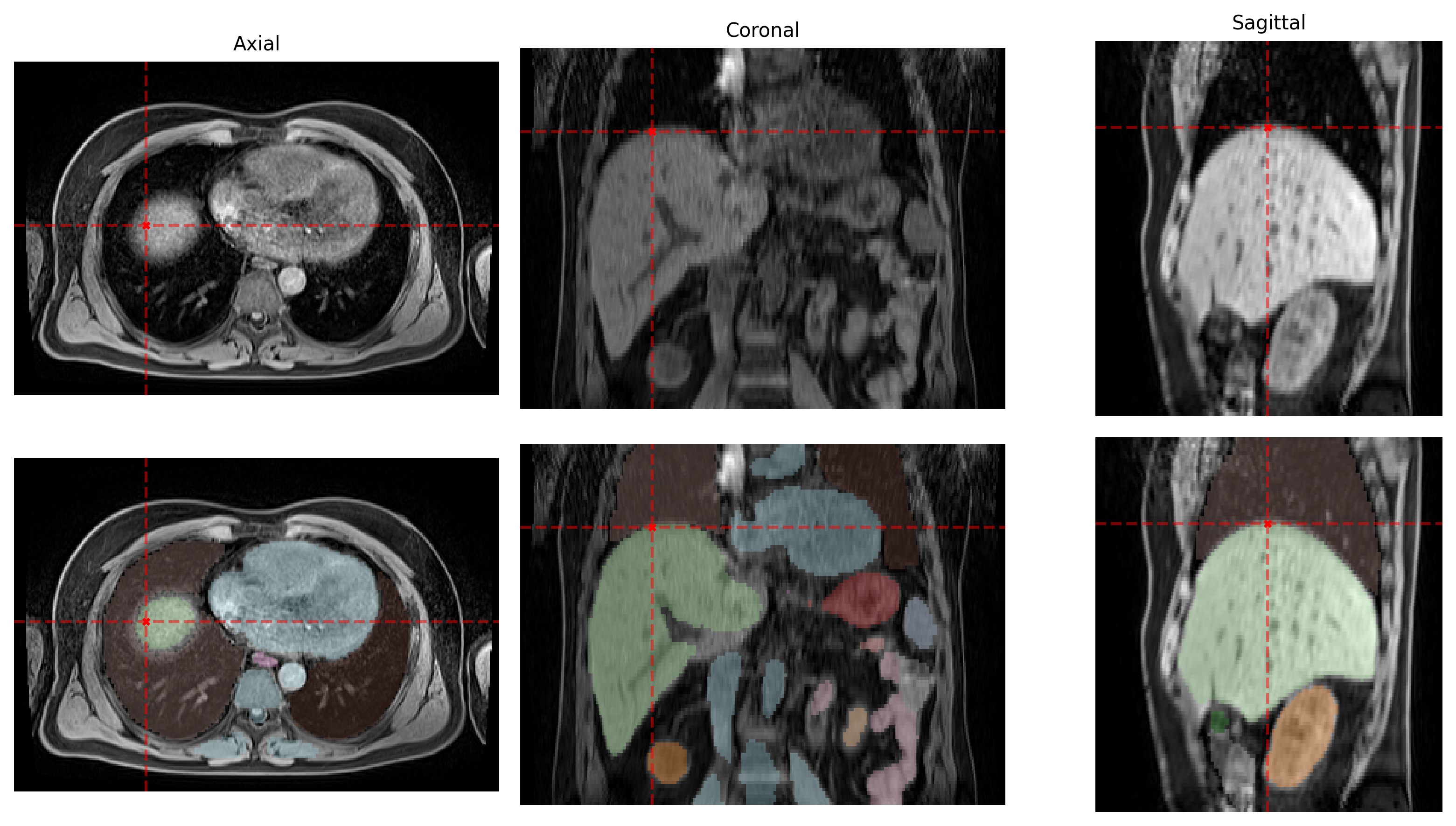

- **Gallbladder Top**: Centre of mass of the gallbladder contour on its most superior slice.
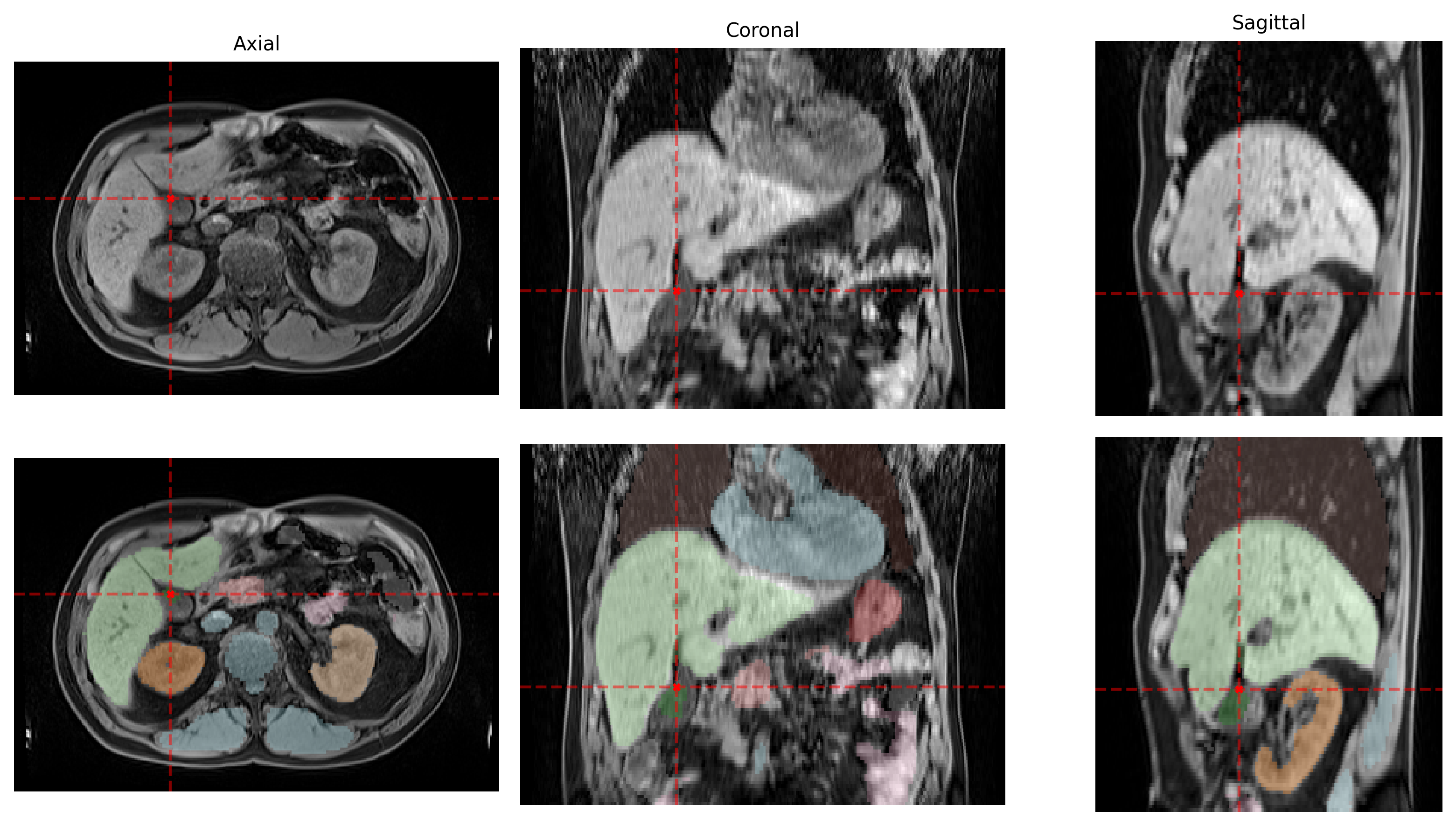

- **Pancreas Top Right**: On the most superior slice containing pancreas tissue, the algorithm identifies the rightmost point of the pancreatic contour.
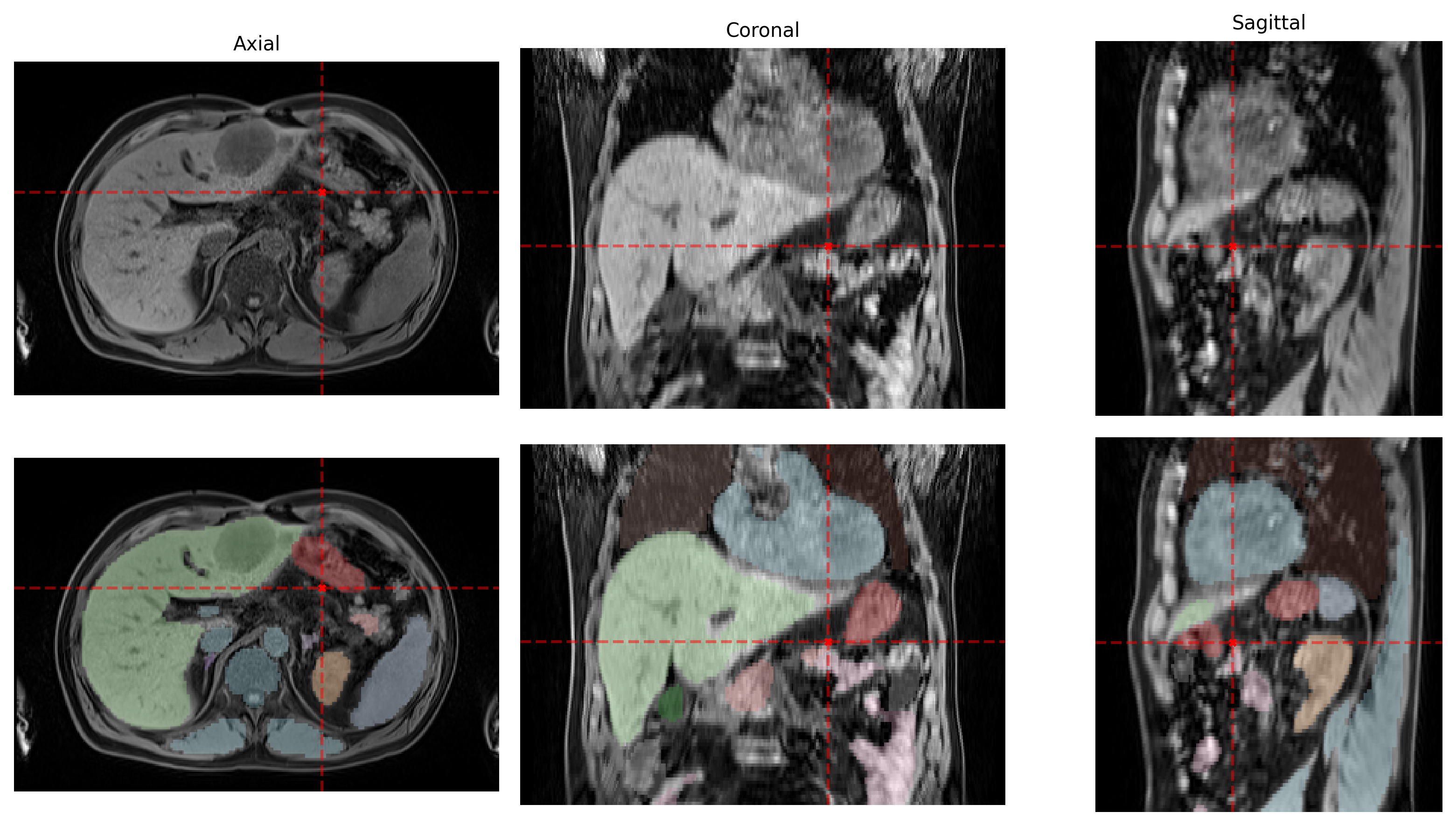

- **Portal Vein Top**: Center of mass of the portal and splenic vein complex on its most superior slice.
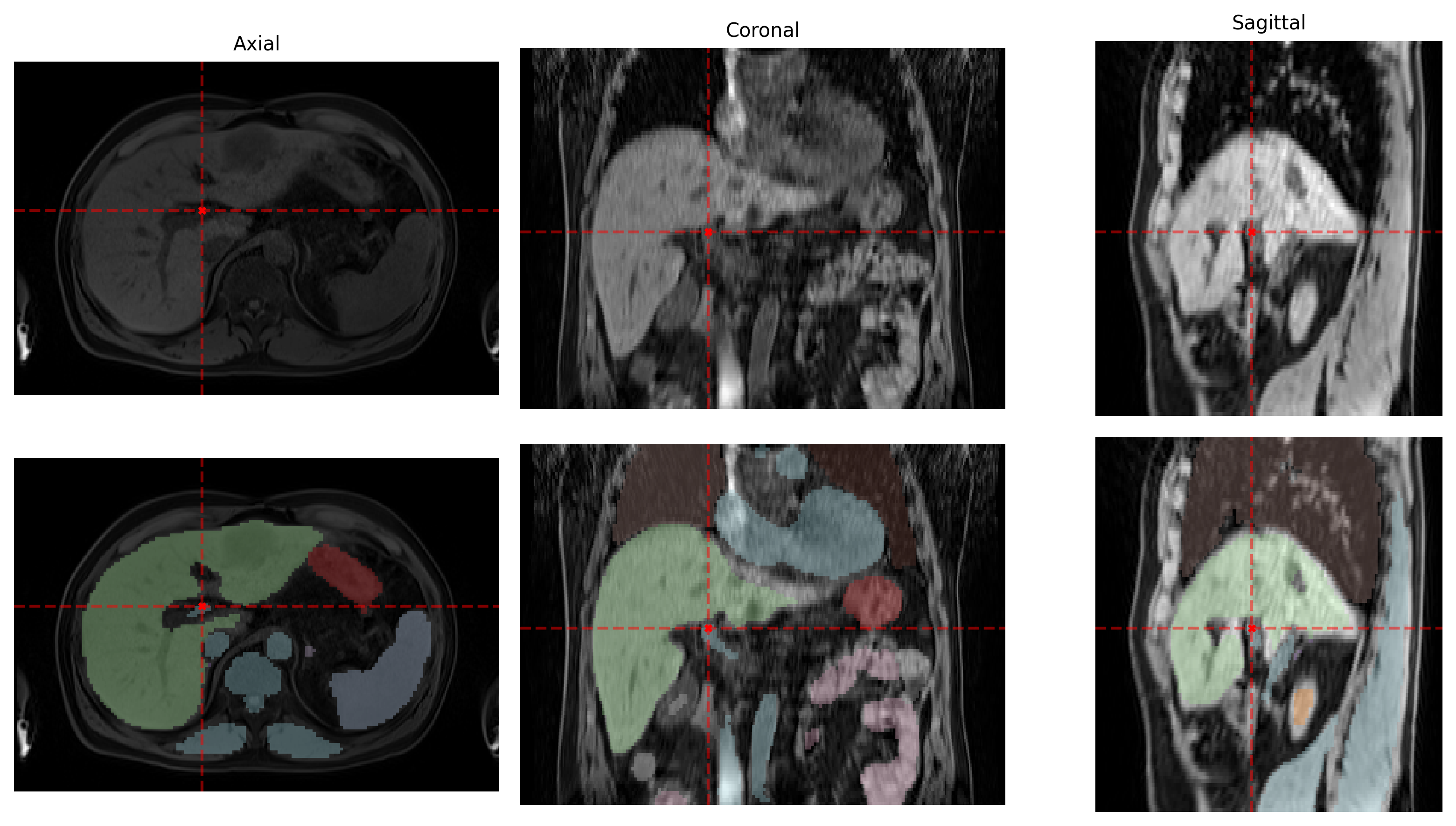

- **Right Kidney Top**: Center of mass of the right kidney contour on its most superior slice.
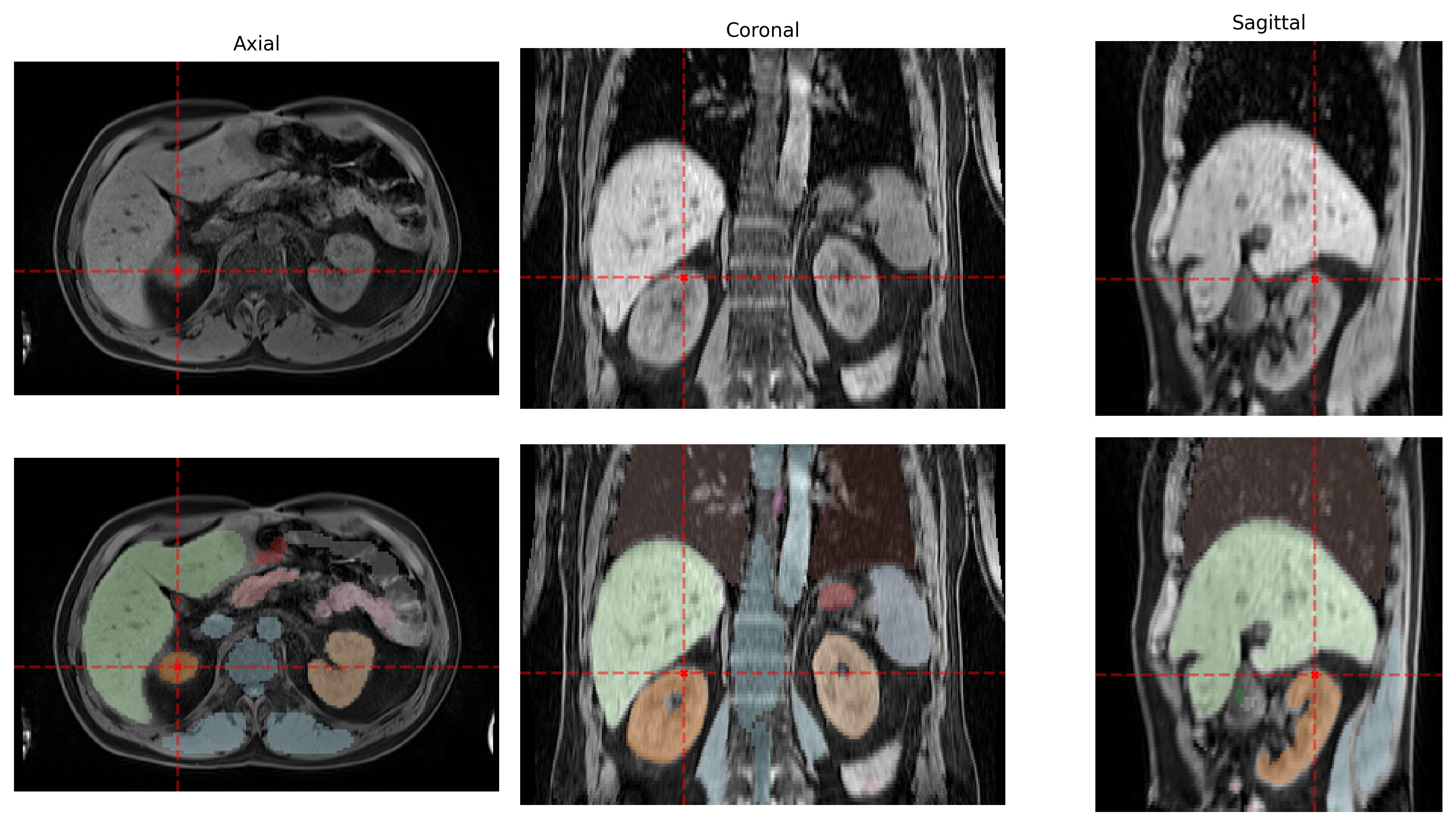

Supplement: Supplementary file 1 — Supplementary Material 1 [file 261_2025_5123_MOESM1_ESM.docx]
